# A systematic review of the concept and clinical applications of Bone Marrow Aspirate Concentrate in Orthopaedics

**DOI:** 10.1051/sicotj/2017007

**Published:** 2017-03-06

**Authors:** Mohamed A. Imam, Samer S.S. Mahmoud, James Holton, Dalia Abouelmaati, Yasser Elsherbini, Martyn Snow

**Affiliations:** 1 Department of Trauma and Orthopaedics, Faculty of Medicine, Suez Canal University 41111 Ismailia Egypt; 2 The Royal Orthopaedic Hospital B31 2AP Birmingham UK; 3 Health Education North East of England NE15 8NY Newcastle UK; 4 Birmingham University B15 2TT Birmingham UK; 5 Research and Development, OxCell OX3 8AT Oxford UK; 6 Institute of Biomedical Engineering, University of Oxford OX3 7DQ Oxford UK

**Keywords:** Bone Marrow Aspirate Concentrate, BMAC, Mesenchymal Stem Cells

## Abstract

*Introduction*: Mesenchymal stem cells (MSC’s) are believed to have multipotent plasticity with the capability to differentiate along multiple cell lineages such as cartilage, bone, tendon, muscle, and nerve. Such multipotency has the potential to play an important role in the repair and reconstruction of multiple tissues across a number of orthopaedic specialties. Bone marrow and fat are the most abundant and accessible source of MSC’s with bone marrow aspirate the most commonly being reported to stimulate healing.

*Methods*: This review examines the current reported 20 Q2 clinical applications of bone marrow aspirate concentrate and its effectiveness.

*Results*: The published studies reported techniques of collection and preparation of BMAC in addition to its applications in a number of orthopaedic sub-specialities. Studies could be sub-categorised into: techniques of extraction, processing and microscopic examination of BMAC (31), reconstruction of osseous defects/non-union (20), treatment of avascular necrosis (9), repair of cartilage defects (8), treatment of sports injuries and tendon injury/repair (9), injection in regenerative therapy (4), treatment of spine conditions (4) including enhancing postoperative fusion and degenerative disc pathology and orthopaedic oncology (4). A few published studies combined the use of platelet-rich plasma (PRP) with BMAC (4) or compared them in different applications (5).

*Conclusions*: BMAC has been used in bone, cartilage and tendon injuries with encouraging results.

## Introduction

Mesenchymal stem cells (MSCs) [1] are believed to have multipotent plasticity with the capability to differentiate along multiple cell lineages such as cartilage, bone, tendon, muscle and nerve [1–6]. Such multipotency has the potential to play an important role in the repair and reconstruction of multiple tissues across a number of orthopaedic specialties. Bone marrow and fat are the most abundant and accessible source of MSCs with bone marrow aspirate the most commonly being reported to stimulate healing [7, 8].

## Bone Marrow composition

The cellular composition of bone marrow aspirate in normal subjects has been studied by Bain using light microscopy [9]. On analysing small volumes (0.1–0.2 mL) in 50 subjects it was found that neutrophils and erythroblasts were the dominant cell type. Neutrophils were lower in males (32.7%) compared with females (37.4%) but erythroblasts were greater in males (28.1%) compared with females (22.5%), which is likely to represent the differences in adult haemoglobin concentrations. The other constituents included lymphocytes 13.1%, eosinophils 2.2%, blast cells 1.4 (immature white cells), monocytes 1.3% and basophils 0.1%. There was a wide variation in the megakaryocyte percentage (platelet precursors) and this is likely to represent the wide variation in normal ranges in the adult population. Yamamura et al. [10] assessed the cellular composition using laser photometry, with the mean subpopulation percentages being consistent with Yamamura et al. [10]*.* Kim et al. found similar percentages using a flow cytometry technique analysing 2 mL of bone marrow [11]. Although the mechanism of cellular analysis differs the percentage of each cell type is largely similar (Table 1) [12].

**Table 1.** Results of cytological analysis of bone marrow aspirate and bone marrow concentrate [12].

	Bone marrow aspirate*	Bone marrow concentrate*	Absolute change*	Relative change†	*p*-Value
Platelet count × 103/μL	31.1	208.3	177	8.7	0.002
White blood-cell count × 103/μL	36.5	267	230	7.4	0.0007
Red blood-cell count × 103/μL)	6774	3156	3617	0.5	<0.0001

* These values are presented as the mean and standard deviation. *N* = 10.

† The relative change is presented as the mean with the 95% confidence interval.

### Bone Marrow harvest

The body has numerous potential areas for bone marrow harvest. Hyer et al. compared the iliac crest, tibia and calcaneus as widely accessible areas and assessed the number of osteoblastic connective tissue progenitor cells [13]. The iliac crest yielded a higher mean concentration of osteoblastic progenitor cells compared to the tibia and calcaneus. Gender, smoking status and diabetes were not predictive of osteoblastic progenitor cell concentration. However, with increasing age there is a reduction in the fibroblast colony forming units (CFU-F) and hence stem cells, which may have implications on efficacy in the elderly population [13].

Bantinic et al. looked at the aspirate from the first 1 mL and subsequent aspirates from the iliac crest. In subsequent samples, the nucleated cell population and CFU level were 3 and 10× lower than the first 1 mL of aspirate [14]. This work has been supported by Muschler et al. highlighting that as the volume of aspirate from the iliac crest increases from 2 to 4 mL the number of MSCs decreases by 50% [15]. This is likely due to the proportion of blood in the subsequent samples diluting the concentration of progenitor cells and colony forming units/stem cells. Muschler et al. [15] and Hernigou et al. [16] have defined the sector rule for the aspiration of marrow from the iliac crest, which is based on safety zones. They studied the anatomy of the iliac bone and its adjacent structures vulnerable to injury by the trocar when it is introduced into the iliac crest. The authors used computed tomography to examine 48 iliac crests in 24 pelvises. They divided the iliac crest into six equal sectors from anterior to posterior direction. The authors studied 480 trocar entry points undertaken by six surgeons among 120 patients. They demonstrated that the sector system consistently envisaged safe and unsafe zones for placing the trocar in the iliac crest. They observed increased risk of breaches on obese patients and this risk is decreased in more experienced surgeons. Ninety-four breaches out of 480 entry points occurred with increased risks observed in the thinner sectors in the iliac crest. Additionally, there is increased risk of injuring the external iliac artery in the four most anterior sectors (1–4) especially in females. On the other hand, posterior sectors were associated with increased risk of sciatic nerve and gluteal vessel injury when the trocar was inserted more than 6 cm into the posterior iliac crest. They concluded that the sector rule is a reliable system to use for bone marrow aspiration (BMA) [16]. Hernigou et al. in a separate study also identified that the use of 10 mL syringes to aspirate bone marrow was superior to 50 mL syringes. In 30 patients the 10 mL syringe aspirations resulted in progenitor cell concentrations on average 300% higher. This was believed to be secondary to a larger negative pressure generated in the 10 mL syringe which preferentially removed bone marrow cells and reduced blood contamination [17].

### Bone Marrow concentration

The main concern in using BMA to stimulate tissue repair/regeneration is that only 0.001% of nucleated cells within BMA are MSCs [9, 18–20]. To address this issue, various protocols have been developed to concentrate the nucleated cell numbers to produce bone marrow aspirate concentrate (BMAC). It is hoped that it would then have the sufficient amount of MSCs needed to provide an effective environment of healing and regeneration [20, 21].

Different techniques have been proposed to concentrate bone marrow aspirate to form BMAC. These include the use of Ficoll density gradients in the laboratory setting and automated, closed centrifugation systems in the clinical setting. The exact mechanism of action of BMAC is currently not fully understood. Potentially the MSCs contained within BMAC will provide a direct cell source for repair of the host tissue. Alternatively or in addition, the nucleated cells may have a significant paracrine effect delivering various cytokines and growth factors to orchestrate and direct host repair [22–25].

This review examines the currently reported clinical applications of BMAC and its effectiveness.

## Methods

### Eligibility

We have included all published clinical literature investigating the development, techniques and applications of BMAC. Language, design and risk of bias did not deter the initial inclusion of any study. Our search was exclusively limited to studies involving human subjects.

### Search Strategy

A PRISMA-compliant search was carried out as published in 2009 [26]. This included the online databases: PubMed, EMBASE, ClinicalTrials.gov and the Cochrane library from 1960 to the end of May 2015. The Medical Subject Heading (MeSH) terms used included: “Bone” AND “Marrow” AND “Aspirate” AND “Concentrate”.

### Critical Appraisal

Eligible studies were independently appraised by two authors using the Critical Appraisal Skills Program checklist. For the purpose of narrative review, relevant studies were included irrespective of methodology or level of evidence.

## Results

Eighty-nine of the 122 (58 PubMed and 64 EMBASE) results yielded by the preliminary search were included. Exclusions included seven duplicate records, four letters, 22 non-orthopaedics related studies (11 vascular surgery, 10 maxillo-facial and one oncology) and two records irrelevant to our search topic.

On searching www.ClinicalTrials.gov, we found that 28 trials were registered. All trials examined the use of stem cells in different orthopaedic applications (Table 2). The CASP appraisal confirmed a satisfactory standard of all 89 studies [27].

**Table 2:** Clinical trials of stem cells and orthopaedic applications registered at ClinicalTrials.gov (June 2015).

Phase	Cell type	Status	Condition	Number enrolled	Institute	Completion	Access No.
	Stem cells	Enrolling	Hip arthroplasty	50	University of Nebraska	April 2012	NCT01366911
II	MSCs	Recruiting	Knee cartilage injuries	16	University of Jordan	June 2015	NCT02118519
II/III	Autologous MSCs	Unknown	Articular cartilage defects	25	Cairo University	December 2014	NCT00891501
II	Autologous MSCs	Not recruiting	Achilles tendinopathy	10	University College London	May 2016	NCT02064062
II	Allogeneic MSCs	Unknown	Osteoarthritis	60	Stempeutics Research Pvt Ltd	July 2014	NCT01453738
0	Allogeneic UCMSCs	Enrolling	Osteochondral lesion of talus	28	Samsung Medical Centre	August 2015	NCT02338375
I/II	MSCs	Completed	Meniscectomy	55	Mesoblast	April 2008	NCT00225095
I	MSCs	Unknown	Bone healing	5	University of Indonesia	December 2014	NCT01725698
II	MSCs	Recruiting	Hip avascular necrosis	30	Universidad Autónoma de Madrid	February 2017	NCT02065167
I/II	Autologous MSCs	Unknown	Chondral knee defects	30	Fundación para la Investigación Biomédica del Hospital Universitario La Paz	June 2012	NCT01399749
II	Allogeneic MSCs	Recruiting	Tibial closed diaphyseal fractures	40	Royan Institute	December 2015	NCT02140528
I/II	MSCs	Active, not recruiting	Knee osteoarthritis	30	University of Navarra	November 2014	NCT02123368
		Enrolling	Posterolateral lumbar fusion	60	University of Utah	January 2016	NCT01409954
	Autologous MSCs	Completed	Fracture non-union healing	35	Keele University	October 2011	NCT02177565
I/II	MSCs/PRP	Recruiting	OA knee	24	Aditya K Aggarwal	June 2014	NCT01985633
I	Autologous MScs	Completed	Ankle joint osteoarthritis	6	Royan Institute	September 2011	NCT01436058
III	BMMSCs	Recruiting	Talar osteochondral lesion	140	Istituto Ortopedico Rizzoli	June 2015	NCT02005861
I	BMMSCs	Completed	Bone cyst	6	Royan Institute	October 2011	NCT01207193
II/III	MSCs	Withdrawn	Benign bone lesion	0	Emory University	March 2010	NCT00851162
	Allogeneic MSCs	Active, not recruiting	Subtalar arthrodesis	140	AlloSource	February 2017	NCT01413061
I/II	Autologous MSCs	Active, not recruiting	Enhance bone healing	30	Institut National de la Santé et de la Recherche Médicale, France	November 2015	NCT01842477
III	Autologous osteoblasts	Recruiting	Osteonecrosis	130	Bone Therapeutics S.A.	June 2017	NCT01529008
II/III	Autologous MSCs	Recruiting	Non-union treatments	60	Royan Institute	August 2017	NCT02448849
	MSCs	Unknown	Osteoarthritis	30	University of Dresden	December 2010	NCT01038596
II	MPCs	Active, not recruiting	Lumbar back pain	100	Mesoblast	July 2015	NCT01290367
I/II	Autologous MSCs	Recruiting	Knee OA	12	University of Toronto	February 2021	NCT02351011
III	Allogeneic MSCs	Recruiting	Chondrogenic discogenic lumbar back pain	330	Mesoblast	December 2017	NCT02412735
II	MSCs	Active, not recruiting	Lumbar interbody fusion	24	Mesoblast	July 2015	NCT00996073

The published studies reported techniques of collection and preparation of BMAC in addition to its applications in a number of orthopaedic sub-specialities. Studies could be sub-categorised into: techniques of extraction, processing and microscopic examination of BMAC (31), reconstruction of osseous defects/non-union (20), treatment of avascular necrosis (9), repair of cartilage defects (8), treatment of sports injuries and tendon injury/repair (9), injection in regenerative therapy (4), treatment of spine conditions (4) including enhancing postoperative fusion and degenerative disc pathology and orthopaedic oncology (4). A few published studies combined the use of platelet-rich plasma (PRP) with BMAC (4) or compared them in different applications (5).

### Literature review

Hernigou et al. highlighted that the efficacy of BMAC is dependent on the amount of progenitor cells in the concentrate. They compared the quantity and concentration of these cells in both BMA and BMAC aspirated from the iliac crest when used for the treatment of atrophic non-union of the tibia. The BMA contained a mean of 612 ± 134 compared to 2579 ± 1121 cells per cm^3^ in the BMAC group. Reduced concentration of progenitor cells at the non-union sites was significantly associated (*p* < 0.01) with non-union [28].

Different techniques have been proposed to concentrate the marrow aspirate to form BMAC. These include the use of Ficoll density gradients in the laboratory setting and automated, closed centrifugation systems in the clinical setting. Although these techniques increase the number of MSCs, they do not significantly increase the ratio of stem cells to other nucleated cells [20, 21, 29]. Centrifugation is the present technique of choice for the various commercially available products used in the clinical setting, although as shown in relation to PRP there is significant variation in the final end products achieved. Fortier et al. compared the constituents of PRP and BMAC, there are reduced platelets and raised white blood cells (WBCs) in BMAC demonstrating that this is a very different substance to PRP with a likely different mechanism of action [12].

### Bone defects

Existing common approaches to managing bone defects involve using allograft bone or harvesting bone from a donor site, usually iliac crest. BMAC has been used to augment this approach with the aim of improving the incorporation of bone graft and in some cases replace it. BMAC utilisation in managing bone defects has been popularised as MSCs can differentiate into osteoblasts and are able to promote osteogenic differentiation in vitro without any osteogenic stimuli [21–23, 28, 30].

Bone Marrow Aspirate Concentrate was used in the treatment of atrophic non-union in 60 patients by Hernigou et al. [28]. They reported a positive association between the quantity of hard callus and the number (*p* = 0.04) and concentration (*p* = 0.01) of fibroblast colony-forming (FCF) units in the graft. In the seven non-united tibias, the concentration (*p* = 0.001) and the total number (*p* < 0.01) of progenitors cells injected were significantly lower than in those that united. They also reported the time interval needed to achieve union was negatively correlated with the FCF units’ concentration at the site of the graft (*p* = 0.04).

Lee et al. [22] demonstrated superior bone healing in a randomised trial using BMAC with PRP injections during distraction osteogenesis of the tibia in 20 patients (40 tibias). They compared patients receiving an osteotomy site injection of BMAC and PRP (treatment group) versus no injection (control group). The mean cortical healing indexes were significantly higher in the treatment group (*p* < 0.001). Although callus profile and type were not different between the two groups, full weight bearing was allowed earlier in the treatment group than in the control group (index: 0.99 months/cm and 1.38 months/cm, respectively, *p* < 0.001). The main limitation with this study is that they did not evaluate whether the combination of BMAC or PRP has synergistic effects on bone regeneration. This query was addressed by Kassem [31] and Hernigou et al. [32], who revealed that the injection of BMAC alone is efficient in the management of non-union.

The use of BMAC with bone defects has clear potential for use in non-union and distraction osteogenesis. However, further trials are required to refine indications and establish a standardised methodology of preparation and application.

### Avascular necrosis

The use of BMAC in avascular necrosis (AVN) [19, 21, 33–38], particularly of the femoral head [19, 36, 37, 39–42] has been described by a number of authors. In 2002, Hernigou and Beaujean [19] were the first to describe a protocol for BMAC injection combined with conventional core decompression (CD) to manage AVN of the femoral head. They studied 189 hips in which BMAC obtained from the iliac crest was inserted into the necrotic area within the femoral head. They reported excellent outcomes in hips at the pre-collapse stage, with total hip replacement (THR) only warranted in 9/145 (6.2%) of the hips studied at five years follow-up.

The same cohort of patients was evaluated in a more recent retrospective analysis [41], 94 of 534 (17%) hips needed a THR at a mean follow-up of 13 years. Of these, 69 hips (18%) demonstrated complete resolution of the necrotic area on magnetic resonance imaging (MRI). These findings are similar to those reported by Martin et al. who used BMAC and PRP following minimally invasive CD of the femoral head (FH) for AVN in 77 hips [43].

Hendrich et al. [44] used autologous BMAC in the management of AVN in 37 femoral heads and 32 areas of AVN in other locations. The study also included 12 non-unions and 20 other bone defects. The injection of BMAC was performed as part of a CD. They reported that 84/101(83%) of patients were highly satisfied, 7/101(6.9%) moderately satisfied and 1/101(1%) expressed poor satisfaction after a mean follow-up period of 14 months (2–24 months). In their opinion, the preparation of the BMAC within the operating theatre was a viable one-step autologous cell therapy for bone regeneration [44].

In a prospective randomised controlled trial undertaken by Sen et al. [45], 51 hips with AVN in 40 patients were randomly divided into two treatment groups. Twenty-six hips were managed with CD and 26 hips were treated with BMAC injection. The Harris Hip Score and mean hip survival were significantly better in the BMAC group (*p* < 0.05). In the largest study carried out by Zhao et al., 100 patients were recruited. Fifty-one hips underwent CD while 53 hips were managed by BMAC injection. In the CD group, 10/51 (20%) of hips compared to 2/53 (3.7%) of hips in the BMAC group collapsed and finally required a second surgery [45].

In summary, BMAC injection yields satisfactory outcomes in AVN of the femoral head, early diagnosis and treatment prior to bone collapse are linked with better results.

### Cartilage defects

The repair of cartilage defects in animal models is well established. This has been extended to human studies with Gobbi et al. [46] using a single-step technique with BMAC placed on a collagen matrix in 15 patients treated for grade 4 cartilage defects in the knee at 24 months follow-up. Patients showed significant improvement in Visual Analogue Scale (VAS), International Knee Documentation Committee score (IKDC) and Knee injury and Osteoarthritis Outcome Score (KOOS) at final follow-up (*p* < 0.005). Superior outcomes were reported in patients with solitary cartilage defects and those with small lesions. MRI and histology confirmed hyaline-like tissue coverage of the lesions.

The use of BMAC as a single-step technique for the reconstruction of cartilage defects of the talus has been compared to open or arthroscopic autologous chondrocyte implantation (ACI) by Giannini et al. [47]. Eighty-one patients with an average age of 30 ± 8 years were reported. In both treatment groups, a hyaluronic acid membrane was used. A second look arthroscopy was undertaken in all patients with a biopsy at 12 months postsurgery. For all groups the mean American Orthopaedic Foot and Ankle Society (AOFAS) score improved significantly (*p* < 0.0005) at an average of 59.5 ± 26.5 months. There were no significant differences in the change of AOFAS scores between the three groups. Histological evaluations emphasised the formation of type II collagen and proteoglycan expression. However, BMAC provided the advantage of permitting a noticeable decrease in morbidity as a “one-step” technique.

A nonrandomised prospective comparative trial by Gobbi et al. [48] compared the outcome of matrix-induced autologous chondrocyte implantation (MACI) versus BMAC implantation, using the same hyaluronic scaffold, in the patellofemoral joint at a minimum of three years postsurgery. They reported no adverse or postoperative surgical complications in either group. The two groups demonstrated significant improvement in the IKDC, KOOS as well as the VAS, and Tegner score (*p* = 0.001), the IKDC score in the BMAC group demonstrated significantly superior compared to MACI (*p* = 0.015). Trochlear lesions displayed superior outcomes when compared with the patellar lesions in the MACI group. This observation was not found in the BMAC group, as the location was not a prognostic factor. Complete filling of the defect on MRI was 76% in the MACI group compared to 81% in the BMAC group [48].

There is a clear need for future research to review the long-term outcomes of autologous chondrocyte transplantation versus bone marrow derived MSC in the form of BMAC. Short-term outcomes look very promising and may enhance established techniques.

### Tendon injury

Tendon injuries are common musculoskeletal presentations and the potential regeneration and repair properties of BMAC have been explored with promising results. A case series reported by Ellera Gomes et al. [49] reviewed 14 patients (nine women, five men) with full thickness rotator cuff tears managed with transosseous sutures augmented with BMAC utilising a mini-open technique. All patients were followed up for a minimum of one year. They reported significant improvement in the University of California, Los Angeles (UCLA) score improving from 12 ± 3.0 to 31 ± 3.2 at 12 months postsurgery. There were no re-tears reported, however six patients (42%) had high-signal intensity at the critical zone. One revision was reported at two years follow-up.

Hernigou et al. [50] evaluated the efficiency of BMAC compared to that of a matched control group in augmenting arthroscopic single row rotator cuff repair. Patients treated with BMAC demonstrated superior outcomes with improved healing rates and enhanced quality of the repaired tendon, as demonstrated by ultrasound and MRI. Forty-five shoulders (100%) of the BMAC group demonstrated tendon healing by six months, compared to only 30 shoulders (67%) in the control group at the same time point. Furthermore, at ten-year follow-up, the integrity of the repair was maintained in 39 (87%) shoulders in the BMAC group compared to only 20 (44%) in the control group.

A prospective multicentre study by Centeno et al. [51] compared the use of BMAC for the treatment of osteoarthritis (OA) with and without rotator cuff pathology. A total of 115 shoulders were treated with BMAC injection principally for glenohumeral OA with or without a rotator cuff tear. The mean disabilities of the arm, shoulder and hand score [52] and VAS improved significantly from 36.1 to 17.1 (*p* < 0.001) and 4.3 to 2.4 (*p* < 0.001), respectively. These results were consistent with a mean subjective improvement of 48.8%. They reported no significant adverse events at two years follow-up post-surgery.

The use of BMAC in rotator cuff repair has shown initial success in small studies. There is a need for larger scale studies to fully define the potential role of BMAC’s role as an augment and how best to deliver it at the repair site.

## Conclusions

Mesenchymal Stem Cells in BMAC have the potential to self-renew, undertake clonal expansion, and differentiate into different musculoskeletal tissues. MSCs are also known to have an immunoregulatory role and may enhance the normal healing response. BMAC has been used in bone, cartilage and tendon injuries with encouraging results. Alongside well-designed clinical trials, further basic science work is required to investigate the therapeutic action of BMAC. The commercial processing of BMAC needs to be optimised in order to achieve a consistent end product, which will provide predicable and translatable results. The future potential of cell characterisation in order to determine the optimum cell for repair/regeneration of various tissue types also needs to be explored.

## Conflict of interest

The authors declare no conflict of interest in relation with this paper.
